# Are Mental Health, Family and Childhood Adversity, Substance Use and Conduct Problems Risk Factors for Offending in Autism?

**DOI:** 10.1007/s10803-020-04622-0

**Published:** 2020-09-11

**Authors:** Katy-Louise Payne, K. L. Maras, A. J. Russell, M. J. Brosnan

**Affiliations:** 1 Faculty of Health, Education and Society, University of Northampton, Waterside Campus, Northampton, NN1 5PH UK; 2 Department of Psychology, Centre for Applied Autism Research, University of Bath, Bath, UK

**Keywords:** Autism spectrum disorder, Offending, Crime, Mental health, Family and childhood adversity, Substance use, Conduct problems, Risk factors

## Abstract

Mental health difficulties, family and childhood adversity factors, substance use and conduct problems have all been linked to offending behaviour in the general population. However, no large-scale study with comparison groups has investigated these risk factors in relation to autistic offenders. The current research included 40 autistic offenders, 40 autistic non-offenders, 40 typically developed (TD) offenders and 39 TD non-offenders. Conduct problems risk factors differentiated autistic offenders from both non-offender groups (autistic and TD) and mental health risk factors differentiated autistic offenders from both TD groups (offenders and non-offenders). Further research is required to understand more about the role of both conduct problems risk factors in autistic offenders (e.g., age at onset, frequency of behaviours) and the mental health needs of autistic offenders.

## Introduction

Autism spectrum disorder (ASD, hereafter autism) is a neurodevelopmental condition characterised by difficulties with reciprocal social interaction and communication as well as restricted, stereotyped and repetitive behaviour, activities and interests (American Psychiatric Association 2013; World Health Organization 2018). Autism affects approximately 1–3.9% of the general population (Baird et al. 2006; Brugha et al. 2011; Centre for Disease Control and Prevention 2018; May et al. 2017), but is estimated to be up to four and a half times more prevalent in forensic settings (e.g., prisons, secure hospitals) than in the general population (Fazio et al. 2012; Robinson et al. 2012). Within forensic settings, research investigating representative autistic^1^ samples (i.e., total population samples or random samples) and matched comparison groups of typically developed (TD) participants has reported that autistic offenders are more likely to engage in crimes against the person (e.g., sexual offences, assault, robbery; Cheely et al. 2012; Kumagami and Matsuura 2009) and less likely to engage in property crimes (e.g., burglary, arson and trespassing; Cheely et al. 2012; Kumagami and Matsuura 2009). No significant differences have been reported between autistic offenders and TD offenders on public order or drugs offences (Cheely et al. 2012; Kumagami and Matsuura 2009).

The general TD offender literature identifies a number of risk factors for offending, including mental health difficulties (e.g., Bebbington et al. 2017, Murray et al. 2013), family and childhood adversity risk factors (e.g., Derzon 2010; Murray et al. 2012, 2013; Tharp et al. 2012), substance use (e.g., Bebbington et al. 2017; Fazel et al. 2006; Murray et al. 2013; Tharp et al. 2012; Whitaker et al. 2008) and conduct problems (e.g., Mordre et al. 2011; Young et al. 2016). To date, however, there has been limited research into the role of these risk factors (co-occurring mental health conditions; adverse childhood events; substance use; conduct problems) in the likelihood of autistic individuals engaging in offending. The existing research is often limited by small sample sizes, poorly matched groups or having no comparison groups (e.g., Allen et al. 2008). To our knowledge, no research to date has examined a *wide* range of risk factors in autistic offenders with adequate comparison groups, which is critical for understanding why some autistic individuals offend and in the development of appropriate interventions. The present study aimed to address this gap. Below we review some of the key known risk factors for offending in TD populations (and, where available, autistic populations).

### Mental Health Risk Factors

A substantial body of research shows an increased prevalence of mental health issues within TD offender populations (e.g., Fazel and Seewald 2012; James and Glaze 2006; Way et al. 2008; Roberts et al. 2008; Steadman et al. 2009). Indeed, UK-based findings indicate that only 10% of offenders do *not* meet the diagnostic criteria for at least one mental disorder (Bebbington et al. 2017) with depression, personality disorder, anxiety and psychosis being the most commonly reported (Bebbington et al. 2017; Roberts et al. 2008). Within mixed-offence samples of autistic offenders, the reported prevalence of co-occurring psychiatric diagnoses ranges from 13 to 83% (Helverschou et al. 2015; Långström et al. 2009), with personality, psychotic and affective disorders being the most frequently reported (Allen et al. 2008; Helverschou et al. 2015; Långström et al. 2009; Søndenaa et al. 2014). Thus, mental health risk factors appear to play an important role in offending in both TD and autistic populations.

### Family and Childhood Adversity Risk Factors

Meta- and systematic-analyses from TD samples have identified various family and childhood risk factors related to offending, including socio-economic status (SES, Derzon 2010; Murray et al. 2013), childhood abuse (Connolly and Woollons 2008; Cuadra et al. 2014; Hosser et al. 2007; Jespersen et al. 2009; Reckdenwald et al. 2013; Vivolo-Kantor et al. 2013; Wang et al. 2012) and parental violence/family conflict (Tharp et al. 2012). Being placed into foster care (Derzon 2010) and being separated from family have also been identified as risk factors related to TD offending (Derzon 2010). Parental risk factors reported to influence offending include parental psychopathy (Derzon 2010), parental divorce or separation (Derzon 2010), parental antisocial behaviour (Derzon 2010) and parental involvement in crime (Murray et al. 2013). Relatedly, parental incarceration further increases the risk of offending (Murray et al. 2013).

Similarly to the TD literature, autistic offender research suggests that lower family income, separation from family, parental conviction of a violent crime and parental psychopathology increases the risk of violent offending (Heeramun et al. 2017; Helverschou et al. 2015). For example, Helverschou et al. (2015) reported that nearly one third (29%) of autistic offenders had been placed into foster or institutional care. Family violence, physical abuse, sexual abuse and neglect have also been reported to be experienced more frequently by autistic offenders compared to both autistic non-offenders and TD offenders (Kawakami et al. 2012; Kumagami and Matsuura 2009). Whilst early childhood deprivation and trauma can be associated with ADHD symptomology in adulthood, this is not the case for ASD symptomology (Golm et al. 2020). Neglect and physical abuse alone have been reported to increase the likelihood of offending by 6.3 and 3.1 times respectively within a sample of autistic offenders (Kawakami et al. 2012).

### Substance Use

Alcohol and substance misuse are commonly associated with offending, with a substantial body of literature identifying a significant relationship between alcohol use and offending in TD populations across most offence types (see Fazel et al. 2006; Murray et al. 2013; Roberts et al. 2008; Tharp et al. 2012). For example, within a sample of UK offenders, the reported prevalence of alcohol and drug dependence was between 20–32% and 39–55% respectively (Bebbington et al. 2017; Roberts et al. 2008); vastly higher than the 1% substance dependence observed in the general non-offending population (McManus et al. 2016) and the 2% drug dependence and 3% alcohol dependence reported in non-offending autistic populations (Joshi et al. 2013). Similarly, research with autistic offenders has reported that substance use played a role in offending behaviour in up to 38% of cases (Allen et al. 2008; Helverschou et al. 2015; Långström et al. 2009; Søndenaa et al. 2014).

### Conduct Problems Risk Factors

Conduct problems have been frequently associated with delinquency and offending. Some such behaviours are included within conduct disorder and attention deficit hyperactivity disorder (ADHD) diagnoses. Conduct disorder is characterised by aggressive behaviour, deceitfulness, destructive behaviour or persistent/repetitive violation of the rules, and has been highly associated with delinquency and offending (Boduszek et al. 2014; Mordre et al. 2011; Young et al. 2016). Conduct problems can also present in ADHD, which is characterised by impaired attention and overactivity (American Psychiatric Association 2013; World Health Organisation 2018). However, evidence for an association between offending and ADHD is slightly more mixed. Some research illustrates an increased risk of offending (Gudjonsson et al. 2012; Lundström et al. 2014; Mannuza et al. 1989, 2008; Pratt et al. 2002) whereas others suggests that co-morbid disruptive, impulsive-control and conduct disorder diagnoses are what mediate this increased risk (Mannuzza et al. 2008; Mordre et al. 2011; Scatterfield et al. 2007).

Within autistic offender samples, behavioural difficulties including verbal aggression (88%), physical aggression (75%), destructive behaviour (69%), displaying sexually inappropriate behaviour (69%) and overactivity (38%) have been reported (Allen et al. 2008). Similarly to TD research, a large Swedish population-based autistic cohort (n = 5739), reported that co-occurring ADHD and conduct disorder diagnoses largely explained the increased risk of violent offending risk, and that autism served as a protective factor once ADHD and conduct disorder were taken into account (Heeramun et al. 2017). These risk factors, in addition to later onset psychiatric disorder and substance misuse, were the most important individual predictors of violent offending in autistic offenders (Heeramun et al. 2017). Other co-occurring diagnoses such as psychotic disorder and personality disorder may also partly explain some cases of violent offending in autistic individuals (Heeramun et al. 2017). However, their presence is neither ubiquitous nor unique to autistic offenders: within non-offending autistic populations psychotic disorder is estimated to affect approximately 2–12% (Hofvander et al. 2009; Joshi et al. 2013; Lugnegård et al. 2011; Mukaddes et al. 2010) and personality disorder approximately 48–62% (Hofvander et al. 2009; Lugnegård et al. 2012). Thus, other risk factors must play a causal role.

In sum, mental health, family and childhood adversity, substance use and conduct problems have all been widely shown to increase the likelihood of offending in TD populations. In addition, it is apparent that these risk factors are rarely independent of one another (e.g., relationships between psychopathology and substance abuse, and childhood abuse and psychopathology). This highlights the importance of investigating these risk factors together, within a single sample. Whilst some research has investigated offending in autistic samples, it is limited in scope, examining just a few of the above risk factors, using small sample sizes and/or without matched comparison groups (e.g., age, gender, offence type). The aim of the present study was to investigate a wide range of risk factors within a large sample of autistic offenders, comparing results to three comparison groups (autistic non-offenders, TD offenders and TD non-offenders) in order to provide a more comprehensive understanding of the risk factors for offending in autistic individuals. It was predicted that autistic offenders would have higher scores (indicative of increased exposure or difficulties) in mental health, family and childhood adversity, substance use and conduct problems risk factor sub-scales than both autistic and TD non-offender samples.

## Method

### Participants

Eighty autistic participants (40 offenders; 40 non-offenders) and 79 TD participants (40 offenders; 39 non-offenders) were recruited across England and Wales. Autistic offenders and TD offenders were recruited from four prison establishments, two probation services, one approved premises and two secure hospitals. An a priori power analysis indicated that 159 participants would achieve 80% power for detecting a small effect size employing the statistical significance criterion of 0.05 (Cohen 1992).

To recruit offender participants, approximately 50 premises (prisons, hospitals, and approved premises) were contacted and those included within this study reflect establishments who reported having both willing autistic participants and the resources to facilitate participation. Autistic non-offenders were recruited via the National Autistic Society (NAS) and the Research Autism website. TD non-offenders were largely recruited from recruitment agencies, local council facilities and non-academic departments at the University of Bath. An all-male sample was selected because approximately 95% of the prison population is male in the UK (Ministry of Justice 2019).

Autistic offenders were identified as having a formal autism spectrum disorder diagnosis from Criminal Justice System (CJS) staff. Whilst preferable for the researcher to see these diagnoses, ethics did not allow access to offenders’ diagnostic records to anyone outside of the UK’s (CJS). Although it is a limitation of the research that independent diagnostic assessments were not carried out, CJS staff confirmed that individuals had received an autism diagnosis from a qualified professional. Autistic non-offender participants were recruited from autism-specific pathways and self-reported their diagnoses. Subsequent inclusion criteria were that participants were aged 16 years or older and were deemed to have the capacity to consent (as initially indicated by staff and then determined at the point of informed consent). Exclusion criteria for the study were those without a good understanding of the English language, active psychosis/psychotic illness, head injury and/or untreated epilepsy.

All participants completed the Autism Spectrum Quotient-10 (AQ-10; Allison et al. 2012), a 10-item brief screening instrument for autism, which is recommended as a screen for adults by the UK’s NICE guidelines. TD participants were excluded from the present study if they scored above 6 on the AQ-10, as recommended by Allison et al. (2012). As expected, there was a main effect of group for AQ-10 scores, *F*(3,159) = 35.23, *p* < .01, ηp^2^ = .41, whereby both the autistic offenders and autistic non-offenders had significantly higher AQ-10 scores than both TD offenders and TD non-offenders (*p*s < .01, See Table 1). No other comparisons were significant (*p*s > .108).

**Table 1:** Participant characteristics: mean age, IQ and AQ-10 scores (standard deviations are in parentheses)

	Autistic offender (n = 40)	Autistic non-offender (n = 40)	TD offender (n = 40)	TD non-offender (n = 39)
Age	33.65 (11.37)	31.63 (12.44)	37.33 (15.31)	36.94 (14.17)
FSIQ-2	95.65 (17.49)	100.25 (16.00)	87.68 (14.62)	92.21 (12.38)
AQ-10	6.23 (2.58)	6.35 (2.52)	3.63 (1.31)	2.54 (1.23)

Participants also completed the two sub-test version of the Weschler Abbreviated Scales of Intelligence (WASI II; Wechsler 2011), comprising the vocabulary and matrix reasoning subtests. A one-way ANOVA indicated a main effect of group for full-scale IQ (FSIQ) scores, *F*(3, 159) = 4.86; *p* < .01, ηp^2^ = .09. Autistic non-offenders had significantly higher FSIQ scores than TD offenders (*p* < .01). All other FSIQ comparisons were non-significant (*p*s > .124). Groups did not significantly differ on age, *F*(3, 159) = 1.65, *p* = .181, ηp^2^ = .03 (Table 1).

Participants were asked to self-report their index offences (see Table 2). Fisher’s exact test indicated no significant difference between autistic offenders and TD offenders on the type of offence that was committed (*p* = .329).

**Table 2:** Index offences committed by autistic offenders and TD offender participants

	Autistic offenders n (%)	TD offenders n (%)
Violent offences (including robbery)	12 (30.0%)	12 (30.0%)
Sexual offences	19 (47.5%)	16 (40.0%)
Drug offences	1 (2.5%)	5 (12.5%)
Driving offences	1 (2.5%)	1 (2.5%)
Theft/Burglary	2 (5.0%)	3 (7.5%)
Public order offences	3 (7.5%)	0 (0.0%)
Arson	1 (2.5%)	0 (0.0%)
Fraud offences	0 (0.0%)	1 (2.5%)
Missing	1 (2.5%)	2 (5.0%)
Total	40 (100.0%)	40 (100%)

### Procedure

All participants completed the WASI-II (Wechsler 2011), AQ-10 (Allison et al. 2012), Anti-Social Behaviour Subscale of the Self Report Psychopathy Checklist III (SRP III) (Paulhus et al. 2009) and Offending Risk Factors Questionnaire (ORFQ). Participants were assessed by the first author in a quiet location at a time and location that was convenient for both the participant and the establishment (where applicable). For the offender samples, assessments took place in the prison/probation service location. Testing took between 45 and 90 min.

### Measures

*The Anti-Social Behaviour* subscale of the Self Report Psychopathy Checklist III (SRP III; Paulhus et al. 2009) consists of 16 questions (e.g., I have never been involved in delinquent gang activity; I have never attacked anyone with the idea of injuring them). This measure was used to quantify the anti-social behaviours of the sample. Questions are scored on a five-point Likert scale from strongly disagree to strongly agree. Six questions are reverse scored. Higher scores on the anti-social behaviour subscale are indicative of greater anti-social behaviour. The antisocial behaviour sub-scale demonstrates good alpha reliability (0.69–0.82; Gordts et al. 2017; Paulhus et al. 2009; Sandvik et al. 2012) and moderate convergent validity with both the anti-social facet of the PCL-R (0.66; Sandvik et al. 2012) and the behavioural domain of the Comprehensive Assessment of Psychopathic Personality—Institutional Rating Scale (0.60; Sandvik et al. 2012).

*The Offending Risk Factors Questionnaire (ORFQ)* was developed by the authors for the purposes of this study (see Appendix 1). Higher scores indicate a greater number of events (e.g., mental health diagnosis, substance use) experienced. The questionnaire was informed by the TD offender literature and where available the autistic offender literature. It comprised four subscales:

1. *Mental health risk factors* (presence of a mental health diagnosis; receiving support from mental health services; current and past use of psychiatric medication).
2. *Family and childhood adversity risk factors* (involvement of social services during childhood; being placed into the care of social services; Primary caregiver (PCG) mental health difficulties; PCG alcohol misuse; PCG drugs misuse; PCG convicted of crime; observed violence in family home; death of PCG as child; PCG divorce; extreme financial hardship; being bullied at school; hyperactivity as a child).
3. *Substance use risk factors* (drugs; alcohol).
4. *Conduct problems risk factors* (being bullied others at school; demonstrating physical aggression; verbal aggression; destructive behaviour; sexually inappropriate behaviour; over-activity).

The ORFQ demonstrated good internal consistency (α = 0.86) and the conduct problems sub-scale of the ORFQ demonstrated moderate convergent validity with the anti-social behaviour subscale of the SRPIII (0.51). This was an expected finding because whilst both scales measure delinquent behaviours, the SRPIII subscale measures more extreme antisocial behaviours (e.g., I purposely tried to hit someone with the car I was driving) than the ORFQ. Individual subscales demonstrated at least acceptable internal consistency (mental health α = 0.812; family and childhood adversity α = .747; substance use α = 0.714; conduct problems α = 0.766).

### Data Analysis

Firstly, a one-way analysis of covariance was run with IQ as covariate to identify whether there were significant group differences on anti-social behaviour. Next, a one-way ANCOVA was conducted to identify whether there was a significant difference between the four groups on total ORFQ score. Finally, a multivariate analysis of covariance (MANCOVA) was run to identify the effect of group upon each of the four subscales of the ORFQ. Significant effects were followed up with univariate tests and pairwise comparisons with Bonferroni corrections as appropriate.

### Ethics

Ethical approval was obtained from the University of Bath, Department of Psychology Ethics Committee, the National Offender Management Service and the NHS Research Ethics Committee.

## Results

### Anti-social Behaviour

There was a significant main effect of group on the anti-social subscale of the SRP-III, *F*(3, 156) = 32.54, *p* < .001, ηp^2^ = .39, with both of the offender groups scoring significantly higher (i.e., more anti-social) than the non-offender groups (Autistic offender mean = 35.85, SD = 9.16; TD offender mean = 39.83, SD = 9.92; Autistic non-offender mean = 24.91, SD = 8.34; TD non-offender mean = 23.18, SD = 7.06). All *p*s < .001. No significant differences were found between the offenders (autistic, TD; *p* = .646) or the non-offenders (autistic, TD; *p* = 1.000).

### Offending Risk Factors Questionnaire (ORFQ)

There was a significant effect of group on total ORFQ score, *F*(3, 90) = 8.79, *p* < .001, ηp^2^ = .24. Pairwise comparisons indicated that the autistic offenders reported higher total ORFQ scores than both non-offending groups (autistic non-offenders: *p* = .017; TD non-offenders: *p* < .001). The autistic offenders’ total ORFQ score was also higher than the TD offenders at a borderline level of significance (*p* = .052). All other *p*s > .083.

**Fig. 1:**
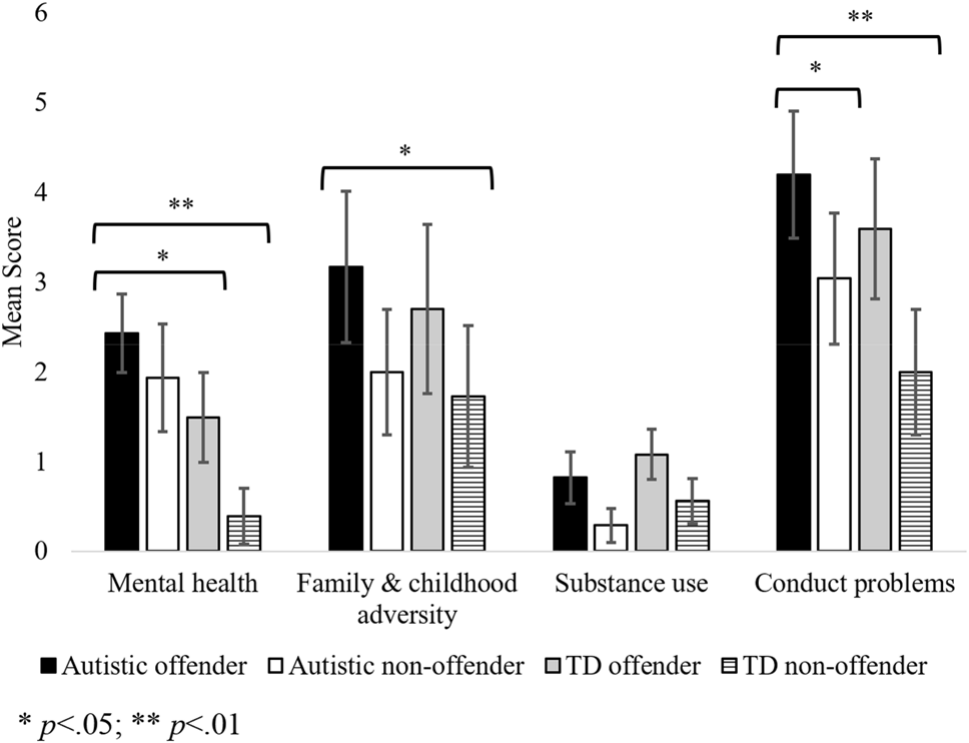
Mean scores for mental health, family and childhood adversity, substance use, and conduct problems risk factors from the Offending Risk Factors Questionnaire (ORFQ) with 95% confidence interval error bars

A MANCOVA including the four ORFQ risk factor sub-scales: (1) mental health; (2) substance use; (3) conduct problems; (4) family/childhood demonstrated a significant overall multivariate effect of group, *F*(12, 255) = 3.13, *p* < .001, ηp^2^ = .13. Univariate tests indicated a significant effect of group for three of the ORFQ risk factor subscales: mental health, *F*(3, 90) = 8.09, *p* < .001, ηp^2^ = .22, substance use, *F*(3, 90) = 3.34, *p* = .023, ηp^2^ = .10, and conduct problems, *F*(3, 90) = 7.71, *p* < .001, ηp^2^ = .21. Univariate tests were at borderline significance for family and childhood adversity, *F*(3, 90) = 2.68, *p* = .052, ηp^2^ = .09. Bonferroni pairwise comparisons for each subscale are described in turn below and illustrated in Fig. 1.

#### Mental Health Risk Factors

The autistic offenders scored higher on the mental health risk factors than both the TD offenders (*p* = .024) and the TD non-offenders (*p* < .001). All other *p*s > .149). ICD-11 classification of mental and behavioural disorders was used to describe the mental health diagnoses reported by the participants (e.g., depression was categorised in mood disorders; anxiety in anxiety or fear-related disorders). A breakdown of specific diagnoses received by group is provided in Table 3.

**Table 3:** Overview of diagnoses reported by participants

ICD-11 Codes	Autistic offender n (%)	Autistic non-offender n (%)	TD offender n (%)	TD non-offender n (%)
Neurodevelopmental disorders (excluding ASD)	7 (17.5%)	1 (2.5%)	2 (5%)	1 (2.6%)
Schizophrenia or other primary psychotic disorders	2 (5%)	0 (0%)	0 (0%)	0 (0%)
Mood disorders	12 (30%)	16 (40%)	12 (30%)	4 (10.3%)
Anxiety or fear-related disorders	8 (20%)	8 (20%)	9 (22.5%)	2 (5.1%)
Obsessive–compulsive or related disorders	3 (7.5%)	0 (0%)	0 (0%)	0 (0%)
Disorders specifically related to stress	3 (7.5%)	0 (0%)	1 (2.5%)	0 (0%)
Disruptive behaviour or dissocial disorder	0 (0%)	0 (0%)	0 (0%)	1 (2.6%)
Personality disorders and related traits	11 (27.5%)	0 (0%)	2 (5%)	0 (0%)

#### Family and Childhood Adversity Risk Factors

Autistic offenders scored significantly higher than the TD non-offender group on family and childhood adversity risk factors (*p* = .039). All other *p*s > .519.

#### Substance Use Risk Factors

No significant group comparisons were found (all *p*s > .054).

#### Conduct Problems Risk Factors

Autistic offenders scored higher than both the autistic non-offenders (*p* = .049) and TD non-offenders (*p* < .001) on the conduct problems risk factors. All other *p*s > .051.

## Discussion

**Fig. 2:**
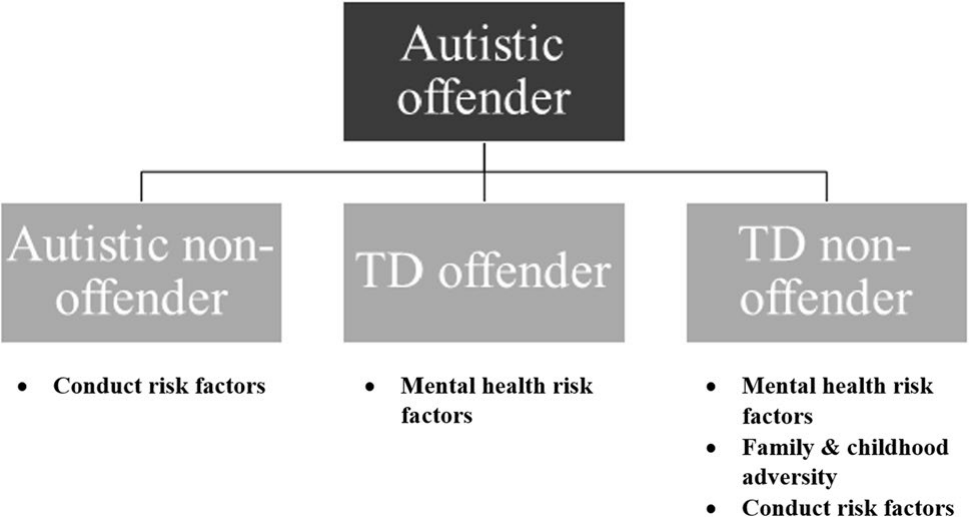
Diagram comparing the risk factors for offending associated with autism versus other comparison groups

The aim of the present study was to investigate risk factors for offending previously identified in TD samples (mental health, family and childhood adversity, substance use and conduct problems) within a large sample of autistic offenders. This study compared autistic offenders to three comparison groups (autistic non-offenders, TD offenders and TD non-offenders) to provide a more comprehensive understanding of the potential risk factors for offending for autistic individuals. Overall, offenders reported more antisocial behaviour than non-offenders, irrespective of autism diagnostic status. However, autistic offenders scored significantly higher on the total number of risk factors on the Offending Risk Factors Questionnaire (ORFQ) overall (i.e., combined mental health, family and childhood adversity, substance use and conduct problems risk factor subscales) than the non-offender groups (autistic and TD) and the TD offenders (borderline significance, *p* = .05). Exploring the subscales of the ORFQ, it was the mental health risk factor that significantly differentiated the autistic offenders from TD offenders and non-offenders, with the autistic offenders scoring higher on the mental health risk factors (but no significant differences between the two autistic groups). Comparing the autistic groups, autistic offenders were, however, significantly differentiated from autistic non-offenders by reporting higher conduct problems risk factors. Autistic offenders were also significantly differentiated from the non-offending TD group by reporting higher risk factors for mental health, conduct problems and family/childhood adversity. Reported substance use was low and did not differentiate the groups, see Fig. 2.

The finding that both offender groups (autistic; TD) scored higher than the non-offender groups (autistic; TD) on the SRP-III anti-social subscale is in line with previous TD offender research (Mordre et al. 2011; Roberts et al. 2008; Young et al. 2016). The current study indicates that the autistic offenders demonstrate increased anti-social behaviours compared to the non-offending samples similar to TD offenders.

The significantly higher total ORFQ scores reported by autistic offenders compared to both non-offender groups indicates that the autistic offenders exhibited and experienced greater numbers of risk factors for offending. The non-significant differences that were reported between the TD offenders and the non-offender groups on the ORFQ total score and its subscales may reflect the mixed findings in TD offender research regarding an association between these risk factors and offending (e.g., Gudjonsson et al. 2012; Lundström et al. 2014; Mannuza et al. 1989, 2008; Pratt et al. 2002).

The finding that autistic offenders scored higher on the conduct problems risk factor than the autistic non-offenders is in line with previous research indicating increased physical and verbal aggression, destructive and sexually inappropriate behaviours in autistic offenders (Allen et al. 2008). Previous research has demonstrated that the age at which conduct problems occur may influence their impact on TD adult offending (Newman et al. 2015; Wanklyn et al. 2012). Further research is necessary to identify whether this is also significant in offending in the autistic population. Childhood psychopathology in autistic populations might be more complicated than simply reporting conduct problems. Aggressive behaviour in childhood has been associated with internalising symptoms (such as anxiety; Bartels et al. 2018), and anxiety disorders are highly prevalent in autistic children (van Steensel and Heeman 2017; White et al. 2009) and adults (Hollocks et al. 2019). It is possible, therefore, that the high rates of conduct problems are a manifestation of the co-occurrence of anxiety. Overall however, it is suggested that autistic individuals who exhibit conduct problems may be at greater risk of offending and warrant additional help and support to help prevent offending.

Furthermore, the autistic offenders scored significantly higher on the mental health subscale than the TD offenders (and TD non-offenders) but not the autistic non-offenders. Previous research suggests that mental health problems may affect up to 70–80% of the autistic population (Lever and Geurts 2016; Simonoff et al. 2008) and the significantly higher needs reported by autistic offenders within this study highlights the critical need to address mental health issues, especially within the CJS context. Whilst there is limited research on the mental health of autistic offenders, Heeramun et al. (2017) suggest that psychopathology is a key risk factor in autistic violent offending. Although the current research found no significant difference between autistic offenders and autistic non-offenders regarding whether an individual had received an additional mental health diagnosis, notable differences were observed in the disorders reported. More of the autistic offenders reported having personality disorder (autistic offenders = 12; autistic non-offenders = 0) and ADHD diagnoses (autistic offenders = 7; autistic non-offenders = 1) compared to autistic non-offenders. Once autism was excluded from the ICD 11 neurodevelopmental diagnosis category (see Table 3), the only diagnosis reported by participants was ADHD. Previous TD offender research has implicated personality disorder in violent crime (Craig et al. 2006; Desmarais et al. 2014; Silver et al. 2008) but not in sexual offending (Whitaker et al. 2008). A visual analysis of the data indicated that the autistic offenders demonstrated a similar trend, with nearly 60% of autistic offenders with comorbid personality disorder reporting violent index offences and less than 10% reporting sexual index offences. The finding of increased rates of ADHD in the autistic sample is unsurprising, as rates of 40–70% of ADHD in autistic individuals have been reported (e.g. Joshi et al. 2013, 2017). Heeramun et al. (2017) report that co-occurring ADHD and conduct disorder explained the elevated violent offending in autistic offenders and reported that autism may actually be protective against criminality (see also Payne et al. 2019b).

Limitations of this study include the self-reporting of mental health, family and childhood adversity, substance use and conduct problems. A self-report approach was selected because more often than not research focusses on caregiver or professional reports often omitting the autistic offenders opinion. This research aimed to address this gap and present research from the perspective of the autistic offender. Whilst there is an on-going debate about the use of self-report with autistic samples there is evidence that suggests autistic individuals can successfully complete self-report tasks (e.g., Hesselmark et al. 2015). Difficulties have been observed when autistic individuals are asked to quantify experience (e.g., choosing between agree or strongly agree) or express themselves with reports indicating that individuals can under-report their difficulties (Findon et al. 2016; Hesselmark et al. 2013; Mazefsky et al. 2011; Shalom et al. 2006). Thus, the current research provided individuals with the option of: (1) yes; (2) no; or (3) don’t know. Whilst steps were taken to ensure participants had the best opportunity to provide true responses it might be helpful for future research to include parents or caregivers to obtain details (e.g., about childhood behaviour or experiences), as well as through direct access to psychiatric reports. One specific limitation is that a number of individuals self-reported that they had been diagnosed with a personality disorder but were unsure of the specific personality disorder diagnosis. Access to mental health diagnostic information would enable development upon this research and Heeramun et al. (2017). Heeramun et al. (2017) found that conduct disorder and ADHD explained the offending behaviours however this finding is specific to violent offending in ASD. Future research should include all comorbid mental health diagnoses and conduct research with all offence types (i.e., not just violent offences). Furthermore, the inclusion of a questionnaire such as the Patient Health Questionnaire Somatic Anxiety and Depression Scale (see Kroenke et al. (2010) for review) could provide more indepth self-reported information about the mental health of participants. The penultimate limitation of this study is the researchers were unable to personally check the offenders police records or the ASD diagnostic assessments of the offender participants due to ethical constraints. The research relied on CJS staff to confirm ASD diagnoses. It must be acknowledged that the level of ASD training and expertise of the staff member was not known however the researchers have no specific reason to question any diagnoses. All autistic participants scored above six on the AQ10. A final consideration is that the analyses controlled for a brief measure of IQ which needs to be intrpreted with caution (Huitema, 1980), as autistic non-offenders had significantly higher FSIQ-2 scores than TD offenders.

Findings from the present study indicate that autistic offenders experienced the highest number of offending risk factors compared to the three other groups. Regarding the management of risk, professionals who work with autistic individuals should be aware of the elevated offending risk associated with an increasing number of risk factors as identified by the higher autistic offender total ORFQ score. Awareness of this risk could enable strategies to be put in place to help prevent initial offending. Despite the higher total ORFQ scores in the autistic offenders, no individual risk factor (i.e., mental health, family and childhood adversity, substance use and conduct problems) effectively differentiated the autistic offenders from the other three groups. Mental health risk factors distinguished the autistic offenders from TD offenders. This finding supports previous research by Heeramun et al. (2017) and highlights the need for autistic offenders to be assessed for mental health difficulties upon contact with Criminal Justice System services and provided with appropriate treatment interventions. Heeramun et al. (2017) reported that psychiatric disorders and substance misuse were the most important individual predictors of violent offending in autistic offenders. Thus suggesting that effective assessment and treatment may help to prevent future re-offending.

In conclusion, the autistic offenders had the highest total ORFQ score suggesting that a greater number of risk factors were experienced by this group however no single risk factor effectively distinguished the autistic offenders from the three comparison groups. Future research should consider more autism-specific risk factors to try to identify differences between autistic individuals who may offend and those who may not. A mixed methods approach could offer great benefit; for example, by employing a qualitative approach to identify reasons for offending, with further objective investigation using quantitative methods (see Payne et al. 2019a). With increased knowledge and understanding of the risk factors for offending (e.g., co-occurring personality disorder or ADHD), those at high risk who have a diagnosis of autism can be provided with more tailored support with a view to preventing autistic individuals from offending. Crucially, such support may need to target the symptomology of the risk factors rather than autism itself. Finally, it is important to note that the majority of autistic individuals are law abiding (Frith 1991; Lundström et al. 2014; Murrie et al. 2002; see also Payne et al. 2019b) and it would be helpful for future research to investigate the reasons why most autistic individuals desist from offending, despite having some of the reported risk factors for offending (see Payne et al. 2019b).

## Offending Risk Factors Questionnaire (ORFQ)

**Table:**

*Mental health*
Have you previously been diagnosed with a mental disorder (e.g., anxiety, depression, personality disorder)? If yes, please list the diagnoses and whether this diagnosis is still applicable
Are you currently prescribed any psychiatric medication (e.g., antipsychotic, antidepressant)?
Have you previously been prescribed any psychiatric medication (e.g., antipsychotic, antidepressant)?
Have you previously received support from mental health services?
*Family and childhood adversity*
Were social services ever involved with you/your family during your childhood? If yes, what for?
Were you placed into the care of social services (e.g., foster placement) during your childhood as a result of the above social service involvement?
Did your parent(s) or primary care giver experience mental health difficulties?
Did your parent(s) or primary care giver misuse alcohol?
Did your parent(s) or primary care giver misuse illegal drugs?
Were your parent(s) or primary caregiver convicted of any criminal offences?
Did you observe any violence within the family home?
Did you experience the death of a parent or primary care giver when you were a child?
Did you experience parental/primary care giver divorce as a child?
Did your family suffer extreme financial hardship when you were a child?
Were you bullied by others at school?
*Substance use*
Have you taken illicit drugs?
Have you frequently excessively drank alcohol?
*Conduct problems*
Did you bully other people at school?
Have you ever been physically aggressive to another person?
Have you ever been verbally aggressive to another person?
Have you ever behaved destructively (e.g., breaking things)?
Have you behaved in a sexually inappropriate way?
Are you over-active (e.g. find it hard to sit still, fidget a lot, always moving and on the go)?
Were you ever described as having a tendency to be hyperactive as a child?

## Footnotes

1 This paper will use identity first language when referring to individuals diagnosed with ASD. The term ‘Autistic’ was the preferred term by many autistic individuals within the UK when asked to choose one term to describe themselves. However, we are respectful that this opinion is not unanimous within the autistic community (see Kenny et al., 2016).
